# Powdered black cumin seeds strongly improves serum lipids, atherogenic index of plasma and modulates anthropometric features in patients with Hashimoto’s thyroiditis

**DOI:** 10.1186/s12944-018-0704-x

**Published:** 2018-03-27

**Authors:** Mahdieh Abbasalizad Farhangi, Parvin Dehghan, Siroos Tajmiri

**Affiliations:** 1 Drug Applied Research Center, Tabriz University of Medical Sciences, Tabriz, Iran; 2 Nutrition Research Center, Tabriz University of Medical Sciences, Tabriz, Iran; 3 Student Research Committee, Tabriz University of Medical Sciences, Tabriz, Iran

**Keywords:** Hashimoto’s thyroiditis, *Nigella sativa*, Black cumin, Lipid profile, Glucose homeostasis

## Abstract

**Background:**

Hashimoto’s thyroiditis is associated with serious alterations in serum lipids and glucose homeostasis. The aims of the current study were to evaluate the effect of powdered *Nigella sativa* on serum lipids, glucose homeostasis and anthropometric variables in patients with Hashimoto’s thyroiditis.

**Methods:**

Forty patients with Hashimoto’s thyroiditis, aged between 22 and 50 years old, participated in the trial and were randomly allocated into two groups of intervention and control receiving powdered *Nigella sativa* or placebo daily for 8 weeks. Serum lipids, glucose homeostasis, and anthropometric variables were evaluated at baseline and after intervention.

**Results:**

Treatment with *Nigella sativa* significantly reduced body weight and body mass index (BMI). Serum concentrations of low density lipoprotein cholesterol (LDL) and triglyceride (TG) also decreased in *Nigella sativa*-treated group after 8 weeks; while serum high density lipoprotein cholesterol (HDL) significantly increased after treatment with *Nigella sativa* (*P* < 0.05). None of these changes had been observed in placebo treated group. Serum Nesfatin-1 concentrations was in inverse relationship with serum triglyceride (TG) (*r* = − 0.31, *P* = 0.04).

**Conclusions:**

Giving attention to the potent beneficial effects of powdered black cumin seeds in improving serum lipid profile and anthropometric features in patients with Hashimoto’s thyroiditis, this medicinal plant could be considered as a beneficial herbal supplement alongside with the disease- specific medications including Levothyroxine in management of Hashimoto’s thyroiditis- related metabolic abnormalities.

**Trial registration:**

Iranian registry of clinical trials (registration number IRCT2014090819082N2- Registered 2014-09-29).

## Background

Hashimoto’s thyroiditis (HT) is one of the most common human autoimmune diseases and an organ-specific T-cell mediated disease that affects the thyroid glands [1, 2]. The disease is ten times more prevalent in women than in men and affects 2% of general population [3, 4]. A significant proportion of patients have asymptomatic chronic autoimmune thyroiditis and 8% of woman (10% of woman over 55 years of age) and 3% of men have subclinical hypothyroidism [5]. Hashimoto’s thyroiditis is associated with serious alterations in composition and the transport of lipoproteins; Hypothyroidism is characterized by hyper-cholesterolaemia and a marked increase in low-density lipoproteins (LDL) and apo-lipoprotein B (apo B) because of reduced fractional clearance of LDL by a reduced number of LDL receptors in the liver [6, 7]. Dyslipidemia occurred in thyroid abnormalities is a potent risk factor of cardiovascular events and myocardial infarction among patients with abnormal thyroid function. Numerous studies revealed that hypothyroidism is an independent risk factor of mortality from cardiovascular disease and all-cause mortality [8, 9]. Moreover, Hashimoto’s thyroiditis is a risk factor of non-insulin dependent diabetes mellitus (NIDDM) and more often these two diseases are in co-existence with each other [10]. Up to 38% of patients with NIDDM have also Hashimoto’s thyroiditis [11].

Considering these metabolic abnormalities in Hashimoto’s thyroiditis, therapeutic approaches in treatment of the disease will be important. Levothyroxine sodium is the treatment of choice for Hashimoto’s thyroiditis however its chronic use is related with cardiac dysfunction, left ventricular hypertrophy [12, 13] and rapid bone loss [14].There are limited data evaluating the effects of vitamins or herbal medications in treatment of thyroid abnormalities [15] and no study was available evaluating the effects of herbal medications in treatment of Hashimoto’s thyroiditis in human. *Nigella sativa*is an amazing herb with a rich historical and religious background; it is one of the medicinal plants and belongs to the Ranunculaceae family [16]. The seeds of the *Nigella sativa* are the source of the active ingredients of this plants; it has considerable health promoting effects including its antioxidant, anti-inflammatory and immune-modulatory properties [16]. Numerous studies have extensively studied therapeutic actions of *Nigella sativa* in treating the disease especially in animal models; while human studies in this filed are scarce [17–19]. Lipid- lowering effects of *Nigella sativa* had been studied in several human diseases including hypercholesterolemia [20], type two diabetes mellitus [21] and coronary artery disease [22]. However, to our review of literature, the effect of this herbal medicine on dyslipidemia or glycemic status and thyroid function among patients with Hashimoto’s thyroiditis has not been evaluated before; therefore in the current study we aimed to test these hypotheses.

## Methods

### Patients

**Fig. 1:**
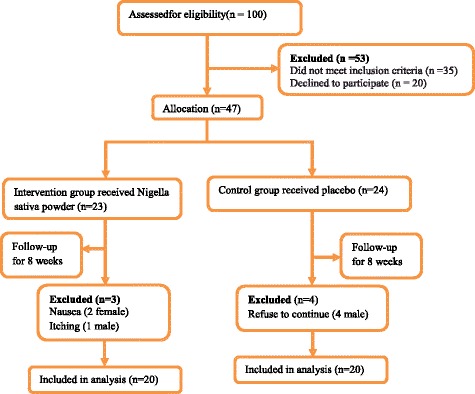
Flow diagram of subject recruitment

In the current double-blinded placebo-controlled trial, forty patients with Hashimoto’s thyroiditis were enrolled (Fig. 1). Subjects were recruited from outpatient endocrinology and metabolism clinics of Isfahan University of Medical Sciences. Inclusion criteria were as follows: age between 20 and 50 years, having Hashimoto’s thyroiditis according to physician diagnosis based on laboratory analysis of thyroid stimulating hormone (TSH), T3, T4 and anti-thyroid peroxidase concentrations. Exclusion criteria were as follows: taking any nutritional supplements for at least 3 months prior participation or during the trial, any history of autoimmune disease, cardiovascular events, other thyroid abnormalities including Grave’s disease, being pregnant or lactating, any history of thyroid surgeries and being on any dietary regimens during and 3 months before recruitment in the trial.

### Study design

From one hundred recruited subjects, fifty three participants were excluded because of not meeting the inclusion criteria or decline to participate. Among forty seven patients random permuted block procedure was performed and participants were randomly allocated into *Nigella sativa*-treated (*n* = 24) or placebo-treated (*n* = 23) groups.

Patients in the intervention group received a daily dose of 2 g *Nigella sativa* powder per day and placebo group received 2 g starches per day for 8 weeks. The mature *Nigella sativa* seeds were obtained from a local market and were milled in a grinder. Both *Nigella sativa* powder and placebo were identically packaged to have similar appearance. Subjects were advised to receive the supplement or placebo packages in two divided dosages with lunch and dinner. Randomization procedure was performed by a third investigator with no clinical involvement in the trial for ensure in blinding. A follow-up procedure was done with weekly telephone contacts to ensure that subjects consumed the supplements regularly.

Written informed consent was obtained from all of the participants before participation in the trial and the study protocol was approved by the ethics committee of Tabriz University of Medical Sciences (Project number: 93173). The current trial was also registered in the Iranian Registry of Clinical Trials (Identifier: IRCT2014090819082N2) [23, 24].

### Anthropometric and nutritional assessments

Body weight and height were measured with a calibrated digital scale and stadiometer respectively. BMI was calculated as weight (kg) divided by height (m) squared [25]. Waist circumference (WC) was measured in horizontal plane, midway between the lowest rib and the iliac crest with a measuring tape in centimeter. Waist to hip ratio (WHR) was calculated by WC divided by hip circumference (HC) [26]. The dietary assessments were performed using a 3-day food record, covering two weekdays and one weekend day, to estimate total energy, carbohydrate, protein, fat and vitamins consumption. Nutrient analysis of the 3-day food record was performed using the Nutritionist IV software (N-squared Computing, Salem, OR, USA).

### Physical activity level

Physical activity was obtained by the questionnaire with nine different metabolic equivalent (MET) scales ranging from sleep/rest (0.9 METs) to high-intensity physical activities (> 6 METs). For each activity level, the MET value was multiplied by the time spent at that particular level. The MET-time at each level was added to obtain a total over 24 h MET-time, representing the physical activity level on an average weekday. Physical activities of different intensities were categorized to sedentary (< 3 METs), moderate (3–6 METs) and vigorous (> 6 METs) respectively [27].

### Biochemical assays

Fasting blood samples were obtained from all of the participants at the beginning and end of the trail. The serum and plasma samples were separated by centrifugation at 2500 rpm for 10 min (Beckman Avanti J-25; Beckman Coulter, Brea, CA, USA) at room temperature. The serum samples were stored at − 70 °C immediately after centrifugation until their assays.Serum total cholesterol (TC), fasting serum glucose (FSG), triglyceride (TG), high density lipoprotein cholesterol (HDL-C) and low density lipoprotein cholesterol (LDL-C) were analyzed by enzymatic colorimetric method (Pars – Azmoon, Tehran – Iran). Serum insulin was analyzed with enzyme linked immunosorbent assay method (ELISA- Monobind Insulin AccuBind, CA 92630, USA). The Sensitivity of this assay was 0.75 μIU/ml and mean inter and intra assay coefficient of variations (CV) were < 9.8% and < 8% respectively. Atherogenic index of plasma (AIP) was calculated as log TG divided by HDL-C [28]. Assessment of insulin sensitivity was performed by the homeostasis model assessment of insulin resistance (HOMA-IR) based on fasting glucose and insulin measurements as follows: HOMA-IR: (glucose (mg/dl) × insulin (mU/l)) / 405. High HOMA-IR scores denote high insulin resistance [29]. Serum Nesfatin-1 concentration was also assessed by ELISA method (Hangzhou Eastbiopharm Co, USA). This assay had a sensitivity of 0.15 ng/ml.

### Statistical assays

Statistical analysis was performed by SPSS™ statistical software (SPSS Inc., Chicago, IL, USA). Quantitative data were presented as mean ± standard deviation (SD), and qualitative data were demonstrated as frequency and percent. Kolmogorov-Smirnov test was used to assess the normality of data. Between groups comparisons of continuous variables were performed by independent sample t-test. Paired t-test was used for before and after intervention comparisons. Analysis of covariance (ANCOVA) was used to identify any differences between two treatment groups after intervention adjusting for the confounding effects of baseline concentrations of parameter, age and gender.

## Results

**Fig. 2:**
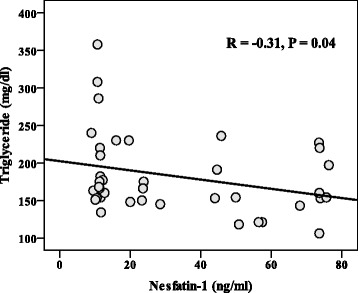
Relationship between serum Nesfatin-1 and triglyceride concentrations in total participants

In the current study 40 patients with Hashimoto’s thyroiditis were enrolled. The demographic and biochemical characteristics of study population are shown in Table 1. Participants in two Nigella-sativa and placebo treated group were similar in their mean age and gender distribution. Baseline values of anthropometric variables were also similar between groups. Whereas, weight and BMI of Nigella-sativa group significantly reduced after 8 weeks intervention period while no changes observed in placebo-treated group. Dietary energy and nutrient intakes before and after intervention are presented in Table 2. Energy and nutrient intakes were similar between groups before intervention and no significant change observed after intervention. *Nigella sativa* significantly reduced serum LDL, TG and AI*P* values (*P* < 0.05). Serum HDL increased after *Nigella sativa* supplementation. None of these changes were observed in placebo treated group. Serum Nesfatin-1 also did not change after intervention; moreover, serum TSH and anti-TPO concentrations reduced while serum T3 increased in *Nigella sativa* treated group (P < 0.05). (Table 3). A significant negative relationship was observed between baseline values of Nesfatin-1 and serum triglyceride concentrations (Fig. 2).

**Table 1:** Demographic characteristics and anthropometric variables in treatment groups before and after intervention

N	Nigella Sativa treated Group	Control group	P^†^
	*N* = 20	*N* = 20	
Age (years)	35.70 ± 8.18	33.95 ± 8.72	0.52
Female [n(%)]	17 (85)	17 (85)	0.89
Weight (kg)			
Before	70.52 ± 12.27	69.63 ± 11.75	0.81
After	69.39 11.84	69.62 11.80	0.95
*P‡*	**0.004**	0.91	
BMI (kg/m^2^)			
Before	27.10 ± 4.63	25.93 ± 4.07	0.40
After	26.63 ± 4.42	25.95 ± 4.11	0.61
*P‡*	**0.002**	0.65	
WHR			
Before	0.86 ± 0.052	0.87 ± 0.053	0.53
After	0.86 ± 0.05	0.87 ± 4.11	0.61
*P ‡*	0.38	0.53	
Physical activity (Met-min/day)			
Before	5.25 ± 0.40	5.29 ± 0.56	0.81
After	5.26 ± 0.43	5.55 ± 0.89	0.20
*P ‡*	0.89	0.22	

Data are presented as mean ± SD or number (percent). *BMI* body mass index, *WC* waist circumference, *HC* hip circumference, *WHR* waist to hip ratio; ^†^*P* values for ANCOVA after adjustment for age, duration of the disease and variable’s baseline value; ‡ *P* values for paired *t*-test

The bold data present statistically significant values

**Table 2:** Dietary intakes of energy and nutrients in treatment groups before and after intervention

N	Powdered Black Cumin Treated	Placebo treated	P
	*N* = 20	*N* = 20	
Energy (kcal/d)			
Before	2251.90 ± 349.58	2208.95 ± 327.80	0.69
After	2236.40 ± 248.27	2265.45 ± 270.73	0.72
*P‡*	0.77	0.32	
Carbohydrate (%)			
Before	57.11 ± 2.90	57.07 ± 3.78	0.97
After	57.24 ± 2.71	57.58 ± 3.32	0.72
*P‡*	0.26	0.67	
Protein (%)			
Before	15.73 ± 1.68	15.07 ± 1.29	0.17
After	15.77 ± 1.36	15.10 ± 1.81	0.19
*P‡*	0.94	0.94	
Fat (%)			
Before	27.34 ± 2.15	26.57 ± 2.32	0.28
After	26.56 ± 1.96	26.13 ± 2.48	0.55
*P‡*	0.51	0.51	
Vitamin E (mg/d)			
Before	2.98 ± 1.08	3.43 ± 1.48	0.36
After	2.89 ± 1.64	3.40 ± 1.19	0.28
*P‡*	0.80	0.96	
Vitamin C (mg/d)			
Before	79.75 ± 22.69	75.67 ± 17.24	0.52
After	78.37 ± 11.84	75.45 ± 15.36	0.59
*P‡*	0.77	0.91	

Data are presented as mean ± SD. ^†^*P* values for ANCOVA after adjustment for age, duration of the disease and baseline concentration of parameter; ‡*P* values for paired *t*-test

**Table 3:** Metabolic parameters and thyroid hormones in treatment groups before and after intervention

N	Powdered Black Cumin Treated	Placebo treated	P
	*N* = 20	*N* = 20	
FSG (mg/dl)			
Before	86.60 ± 8.46	88.10 ± 8.56	0.58
After	84.90 ± 7.01	87.80 ± 6.03	0.16
*P‡*	0.31	0.85	
Insulin (μIU/ml)			
Before	10.62 ± 7.51	7.71 ± 4.12	0.14
After	29.18 ± 19.93	17.30 ± 9.16	**0.023**
*P‡*	**< 0.001**	**< 0.001**	
HOMA-IR			
Before	2.32 ± 1.71	1.67 ± 0.92	0.14
After	6.09 ± 4.06	3.76 ± 2.08	**0.03**
*P‡*	**< 0.001**	**< 0.001**	
HDL (mg/dl)			
Before	41.55 ± 4.67	41.70 ± 6.50	0.93
After	43.75 ± 3.72	40.57 ± 4.87	**0.027**
*P‡*	**0.046**	0.26	
LDL (mg/dl)			
Before	130.65 ± 30.68	105.00 ± 34.48	0.018
After	107.85 ± 36.99	108.90 ± 32.88	0.92
*P‡*	**0.002**	0.06	
TG (mg/dl)			
Before	177.10 ± 34.50	186.00 ± 66.63	0.59
After	156.00 ± 21.92	185.55 ± 74.98	0.11
*P‡*	**0.02**	0.93	
TC (mg/dl)			
Before	183.70 ± 45.72	179.10 ± 43.66	0.74
After	175.10 ± 29.06	180.70 ± 44.95	0.64
*P‡*	0.22	0.55	
AIP			
Before	0.62 ± 0.11	0.63 ± 0.15	0.86
After	0.54 ± 0.08	0.63 ± 0.16	**0.04**
*P‡*	**< 0.001**	0.68	
Nesfatin-1 (ng/ml)			
Before	41.80 ± 28.33	25.86 ± 20.91	**0.049**
After	37.63 ± 5.91	26.75 ± 23.95	0.175
*P‡*	0.34	0.69	
TSH (mIU/l)			
Before	6.42 ± 3.86	8.14 ± 7.28	0.35
After	4.13 ± 2.35	8.27 ± 7.21	**0.02**
*P‡*	**0.03**	0.40	
T3 (mmol/l)			
Before	0.92 ± 0.27	1.18 ± 0.36	0.017
After	1.06 ± 0.34	1.16 ± 0.35	0.39
*P‡*	**0.008**	0.15	
T4 (mmol/l)			
Before	8.07 ± 2.56	7.97 ± 3.11	0.91
After	8.89 ± 1.43	7.63 ± 2.23	**0.04**
*P‡*	0.21	0.32	
Anti-TPO (IU/ml)			
Before	294.55 ± 210.05	278.10 ± 170.77	0.78
After	147.99 ± 158.33	274.30 ± 167.20	**0.01**
*P‡*	**0.019**	0.28	

Data are presented as mean ± SD. ^†^*P* values for ANCOVA after adjustment for age, duration of the disease and baseline concentration of parameter; ‡*P* values for paired *t*-test. *FSG* fasting serum glucose, *TC* total cholesterol, *TG* triglycerides, *HDL* high-density lipoprotein cholesterol, *AIP* atherogenic index of plasma, *TSH* thyroid-stimulating hormone, *T3* triiodothyronine *T4* thyroxine, *TPO* thyroid peroxidase

The bold data present the statistically significant values

## Discussion

Hashimoto’s thyroiditis is accompanied with disturbances in serum lipids and glucose homeostasis. The disease is a known risk factor for hyperlipidemia and diabetes mellitus [6, 10]. The results of the current study provide insight into lipid-lowering effects of *Nigella sativa* in patients with Hashimoto’s thyroiditis. Moreover *Nigella sativa* reduced body weight and BMI in these patients. It is the first trial evaluating the effects of *Nigella sativa* on serum lipids and BMI in patients with Hashimoto’s thyroiditis.

Weight reducing effects of *Nigella sativa* has been observed in previous studies; Zaoui A [30] reported a significant reduction in body weight in rats after 6 weeks treatment with *Nigella sativa* fixed oil (*P* < 0.001). In other study 3 month supplementation with 1.5 g daily powdered *Nigella sativa* in central obese men significantly reduced body weight [31]. In other study in menopausal women also slight and non-significant reduction in body weight was observed after 2 month treatment with 1 g/ day *Nigella sativa* [32]. The anti-obesity effects of Nigella might be explained by increasing mean rates of satiety and fullness [33]. Other possible mechanisms includes reduction in lipid absorption, reduced energy intake, increased energy expenditure, decreased pre-adipocyte differentiation and proliferation, or decreased lipogenesis and increased lipolysis [34].

In fact, other health promising effects of *Nigella sativa* like its hypolipidemic or hypoglycemic effects observed in our study and also previous reports [31, 35] could be explained by these mechanisms; we observed a strong reduction in serum LDL and TG and AIP and increase in serum HDL concentrations (*P* < 0.001 and *P* < 0.05 respectively). A mild non-significant reduction in serum FSG was also observed in *Nigella sativa* treated group. Serum insulin was increased in both groups and comparison of mean difference in serum insulin between groups showed no statistically significant difference.

Although not clear explanation can be attributed to this phenomenon, however, the possible underlying reason can be the direct effects of pro-inflammatory cytokines in the Hashimoto’s thyroiditis against insulin resistance and deteriorating the pancreas’ β-cell function; TSH is a potent stimulator of interleukin (IL)-6, IL-2, C-reactive protein (CRP) and tumor necrosis factor (TNF)-α secretion from adipose tissue in patients with Hashimoto’s thyroiditis [36]. On the other hand, previous studies reporting the beneficial effects of *Nigella sativa* on glycemic status and insulin resistance used higher doses of *Nigella sativa* compared with our study [37, 38] or even more prolonged study duration time [39, 40]. Therefore, the beneficial effects of *Nigella sativa* on glycemic status and insulin resistance in the current dose and the study’s duration have not been observed.

The underlying mechanisms previously suggested for hypolipidemic effects of *Nigella sativa* included an up-regulation of LDL-C molecules through receptor mediated endocytosis [35], decreased dietary cholesterol absorption, stimulation of primary bile acid synthesis and its fecal losses probably contributed to its dietary soluble fibers and sterols [32] and non-enzymatic lipid peroxidation by antioxidant properties of *Nigella sativa* making liver cells more efficient to remove LDL-C from blood by increasing LDL-C receptor densities in the liver and binding to apolipoprotein, apo-B [41–44].

In the current study serum Nesfatin-1 concentrations did not change after *Nigella sativa* supplementation. However, its serum concentrations was in inverse relationship with serum triglyceride concentrations (*r* = − 0.31, *P* < 0.05). Nesfatin-1 is a new anorexigenic hormone which is expressed from several regions of hypothalamus and peripheral tissues, including the adipose tissue, gastric mucosa, pancreatic beta-cells. Recent studies have demonstrated that Nesfatin-1 is negatively related with obesity and insulin resistance [32, 45]. Our finding also confirmed these results. However no change after *Nigella sativa* supplementation was observed in serum Nesfatin-1 concentrations. Probably because of the study’s low sample size.

## Conclusions

We have demonstrated beneficial effects of powdered black cumin in improving serum lipids and reducing body weight in patients with Hashimoto’s thyroiditis. Considering these health-promoting effects of *Nigella sativa*, it can be considered as a beneficial herbal supplement alongside with the disease- specific medications including Levothyroxine in management of Hashimoto’s thyroiditis- related metabolic abnormalities.

## Abbreviations

BMI: Body mass index
HC: Hip circumference
T3: Triiodotyronine
T4, Thyroxine: TG-Ab, thyroglobulin antibody
TPO-Ab: Thyroid peroxidase antibody
TRH: Thyrotropin releasing hormone
TSH: Thyroid stimulating hormone, VEGF, vascular endothelial growth factor
WC: Waist circumference; WHR, waist to hip ratio
